# Identifying QTLs for Grain Size in a Colossal Grain Rice (*Oryza sativa* L.) Line, and Analysis of Additive Effects of QTLs

**DOI:** 10.3390/ijms23073526

**Published:** 2022-03-24

**Authors:** Xuanxuan Hou, Moxian Chen, Yinke Chen, Xin Hou, Zichang Jia, Xue Yang, Jianhua Zhang, Yinggao Liu, Nenghui Ye

**Affiliations:** 1 College of Agriculture, Hunan Agricultural University, Changsha 410128, China; 18854806295@163.com (X.H.); chenyinkede@foxmail.com (Y.C.); 2 Co-Innovation Center for Sustainable Forestry in Southern China & Key Laboratory of National Forestry and Grassland Administration on Subtropical Forest Biodiversity Conservation, College of Biology and the Environment, Nanjing Forestry University, Nanjing 210037, China; cmx2009920734@gmail.com; 3 State Key Laboratory of Crop Biology, College of Life Science, Shandong Agricultural University, Tai’an 271018, China; h15621523656@163.com (X.H.); jiazc973@163.com (Z.J.); xueyang202001@163.com (X.Y.); 4 CAS Key Laboratory of Quantitative Engineering Biology, Shenzhen Institute of Synthetic Biology, Shenzhen Institutes of Advanced Technology, Chinese Academy of Sciences, Shenzhen 518055, China; 5 Department of Biology, Hong Kong Baptist University, Hong Kong 999077, China; jzhang@hkbu.edu.hk; 6 State Key Laboratory of Agrobiotechnology, The Chinese University of Hong Kong, Hong Kong 999077, China

**Keywords:** rice, grain size, QTL, BSA-seq, splicing, additive effects

## Abstract

Grain size is an important component of quality and harvest traits in the field of rice breeding. Although numerous quantitative trait loci (QTLs) of grain size in rice have been reported, the molecular mechanisms of these QTLs remain poorly understood, and further research on QTL observation and candidate gene identification is warranted. In our research, we developed a suite of F_2_ intercross populations from a cross of 9311 and CG. These primary populations were used to map QTLs conferring grain size, evaluated across three environments, and then subjected to bulked-segregant analysis-seq (BSA-seq). In total, 4, 11, 12 and 14 QTLs for grain length (GL), grain width (GW), 1000-grain weight (TGW), and length/width ratio (LWR), respectively, were detected on the basis of a single-environment analysis. In particular, over 200 splicing-related sites were identified by whole-genome sequencing, including one splicing-site mutation with G>A at the beginning of intron 4 on *Os03g0841800* (*qGL3.3*), producing a smaller open reading frame, without the third and fourth exons. A previous study revealed that the loss-of-function allele caused by this splicing site can negatively regulate rice grain length. Furthermore, *qTGW2.1* and *qGW2.3* were new QTLs for grain width. We used the near-isogenic lines (NILs) of these GW QTLs to study their genetic effects on individuals and pyramiding, and found that they have additive effects on GW. In summary, these discoveries provide a valuable genetic resource, which will facilitate further study of the genetic polymorphism of new rice varieties in rice breeding.

## 1. Introduction

Rice (*Oryza sativa* L.) is one of the crucial and staple cereal crops in the global village and provides over 21 percent of the food needed by humankind [1]. There are many advantages (published genome sequence, smaller genome size, high efficiency of genetic transformation technology, and rich genetic resources) of rice, making it one of the most popular model plants for studying plant biology, especially among monocotyledons [2]. In 2050, the global population is forecast to increase to 9.7 billion, and we will require more food to sustainably feed the world [3]. To address the food crisis, it is particularly important to increase the yield of rice and other food crops.

Effective tiller number per plant, grain number per tiller, and grain weight are the key factors controlling rice yield. Grain weight is predominantly controlled by grain shape [4]. To date, several major quantitative trait loci (QTLs) and genes controlling grain size have been cloned and characterized (http://www.gramene.org/; accesed on 12 August 2021), most of which play a role in affecting the yield and appearance quality in rice [1,2]. These QTLs and genes, which encode different kinds of proteins, regulate grain size by affecting cell expansion and proliferation in the seed coat or the growth of the endosperm [5].

It has been reported that there are a few genes that affect grain size by controlling cell proliferation [5]. GW2 is a RING-type E3 ubiquitin ligase that negatively controls grain width by anchoring substrates to proteasomes, to regulate the proteolytic process [6]. *GW5* is an important gene for controlling grain width and regulating the ubiquitin–proteasome pathway [7]. Moreover, GS3 is a part of the G protein signaling pathway, which is associated with rice grain size [8,9]. *qGL3* regulates grain length antagonistically and contains two Kelch motifs that are necessary and sufficient for the negative regulatory function of OsPPKL1 [10]. *SG1* functions in the elongation of seeds, by decreasing cellular proliferation and reducing responses to brassinolides (BRs) [11]. *SMG1* encodes a mitogen-activated protein kinase kinase 4 (OsMKK4), which regulates cell proliferation and grain size in rice. OsMKK4 may act as a linkage factor between the BR and MAPK pathways in grain development [12]. OsMKKK10, OsMKK4, and OsMAPK6 work in a common signaling pathway, to regulate the grain size of rice by enhancing cell proliferation [13]. *OsUBP15/LG1* affects grain width by regulating cell proliferation, and a ubiquitin-specific protease15 that it encodes has deubiquitination activity. The results of genetic analysis indicated that *GW2* and *LG1* have some relationship in affecting rice grain width [14]. All of these genes affect grain size by controlling cell proliferation. In addition, several genes affect grain size by controlling cell expansion [5]. *OsBRI1* (*d61*) encodes a putative BR receptor kinase that has a regulatory effect on rice growth and development [15]. SRS5 is an alpha-tubulin protein and independently controls cell elongation in the BR signaling pathway [16]. *GL7* and its negative regulator together regulate longitudinal cell elongation [17]. GS2/OsGRF4 is a transcription factor that enhances grain weight and yield, primarily by regulating cell expansion [18]. OsmiR396 could not regulate the expression of mutated OsGRF4, and OsGRF4 directly interacts with OsGIF1. Thus, there is an OsmiR396–OsGRF4–OsGIF1 module that regulates grain size [19]. As a plant-specific transcription factor, GLW7 affects cell size by controlling cell expansion in the grain hull [20]. *WTG1* encodes a functional deubiquitinating enzyme and affects cell expansion in spikelet hulls, to determine grain size [21].

Alternative splicing (AS) mainly regulates plant development and stress responses at the posttranscriptional level and is one of the molecular mechanisms through which plants regulate their gene interaction network [22,23]. Omics technologies have been diffusely used to explore plant growth, organization development, and molecular characteristics [24,25,26]; these technologies provide large amounts of information and can be used to look for alternative splicing sites. Genome-wide transcriptome mapping has shown that the range of alternative splicing in plants ranges from 42 to 61%. During different stages of plant development and responses to environmental stress, many genes can produce different alternative splicing products to participate in particular developmental processes and responses to various environments [27]. Previous studies have found that approximately 48% of rice genes show alternative splicing patterns [28]. In rice, AS participates in the circadian clock [29], grain quality [30], grain size [31], and so on. Some important stress-response genes of AS, experienced in response to stress, are conserved in different plant species [32,33,34].

In our study, we compared the whole-genome sequence of CG (a colossal grain rice) with that of Nipponbare and found many differences. Among them, AS may play an important role in the particularly large phenotypic differences between CG and the other japonica rice. Meanwhile, we constructed an F_2_ population using a cross between 9311 and CG, to locate QTLs correlated with grain size. In light of the QTL mapping results, we developed a series of different grain size NILs, by successively backcrossing and selfing CG with 9311 as the recurrent parent. The objective of developing these NILs was to analyze the additive and positive effects on the traits. In the future, the results of the current research will be useful for exploring new genes related to grain size.

## 2. Results

### 2.1. Phenotypic Variation and Correlation Analysis

**Figure 1:**
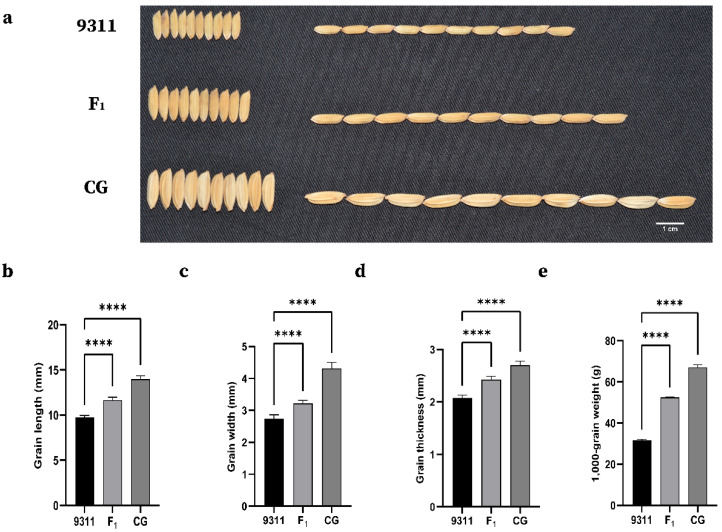
Grain size of two parents and F_1_ individuals derived from the cross between CG and 9311. (**a**) Grain phenotypes of 9311, F_1_, and CG, Bar, 1 cm. (**b**–**e**) Grain length, width, thickness, and 1000-grain weight of 9311, F_1_, and CG. Student’s *t*-test was used to generate *p* values; **** *p* < 0.0001.

**Figure 2:**
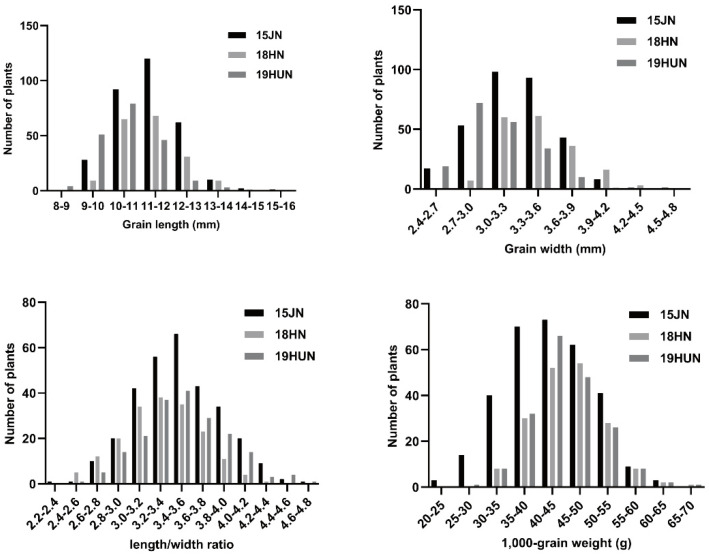
The frequency of the four grain traits in the F_2_ population. The frequency of grain length, width, length/width ratio, and 1000–grain weight in the F_2_ population.

The two parents, 9311 and CG, showed highly significant differences in all grain-related traits (GL, GW, LWR, and TGW). The grain size of CG and 9311 hybrid F_1_ plants was large and full, indicating that the large-grain phenotype was superior to the small-grain phenotype (Table 1; Figure 1). CG had an extra-large grain size per 1000-grain weight (over 65 g) in comparison to 9311 (approximately 32 g). For the other grain-related traits, there was also a significant difference between the parents in all environments (Table 1). This result also indicates that CG can show good yield traits of grain in different environments. All grain-related traits of the F_2:3_ populations exhibited a typical and continuous normal distribution in the three investigated environments (Figure 2), indicating a quantitative inheritance suitable for QTL identification. For all grain-related traits, transgressive inheritance was found in all F_2:3_ populations. Nevertheless, the plants produced by transgressive inheritance exhibited more extreme grain-related traits in Jining, demonstrating that these grain-related phenotypes were potentially regulated by the environment.

To explore the correlations between these traits, we analyzed the Pearson correlation coefficient of the F_2_ population traits using IBM SPSS Statistics 26. GL and GW displayed significant positive correlations with TGW. However, LWR and TGW showed no significant correlations (Table 2).

### 2.2. Identification of TGW QTLs Using QTL-Seq

**Figure 3:**
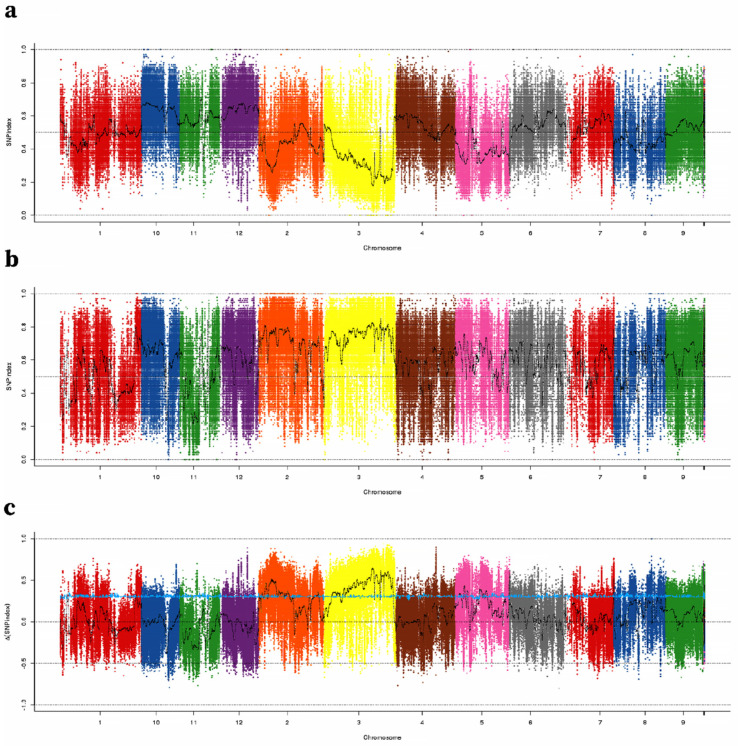
SNP-index Manhattan plot of B-pool, S-pool, and ∆ (SNP-index) from the BSA analysis. SNP-index graphs of (**a**) big-grain bulk and (**b**) small-grain bulk. (**c**) ∆(SNP-index) graph. The *X*-axis shows the position of the 12 chromosomes and the *Y*-axis shows the SNP-index (**a**,**b**) and ∆ (SNP-index). The blue line is the screening threshold at a 95% confidence level. SNPs are set to different colors to help distinguish chromosomal boundaries.

The NGS-based high-throughput sequencing of the two parental genotypes, B-pool and S-pool, resulted in 34,835,652, 31,949,878, 127,452,818, and 110,758,536 high-quality short reads, covering 89.34–96.92% of the Nipponbare reference genome (Table S1). GATK software was used to analyze the SNPs in the results. Finally, we obtained 640,595 high-quality SNPs from polymorphisms in two parents and homozygotes for each parent. The Δ (SNP index) and SNP index were computed using these SNPs. The average SNP index across a 100-kb genomic range was calculated for the S-pool and B-pool using a 1-kb sliding window and mapped by genome location, then Δ (SNP index) was calculated between the two extreme bulk samples (Figure 3). A 95% (blue) confidence level was chosen as the screening threshold, and windows larger than the threshold were selected as our candidate intervals. Three regions were detected on chromosome 2, two regions were detected on chromosome 3, and three regions were detected on chromosome 5. As a result, these regions may contain major QTLs controlling TGW. Nevertheless, these regions contained abundant candidate SNPs, which made it difficult to find candidate genes responsible for TGW.

### 2.3. QTL Mapping of Grain Size in F_2_ Populations

**Figure 4:**
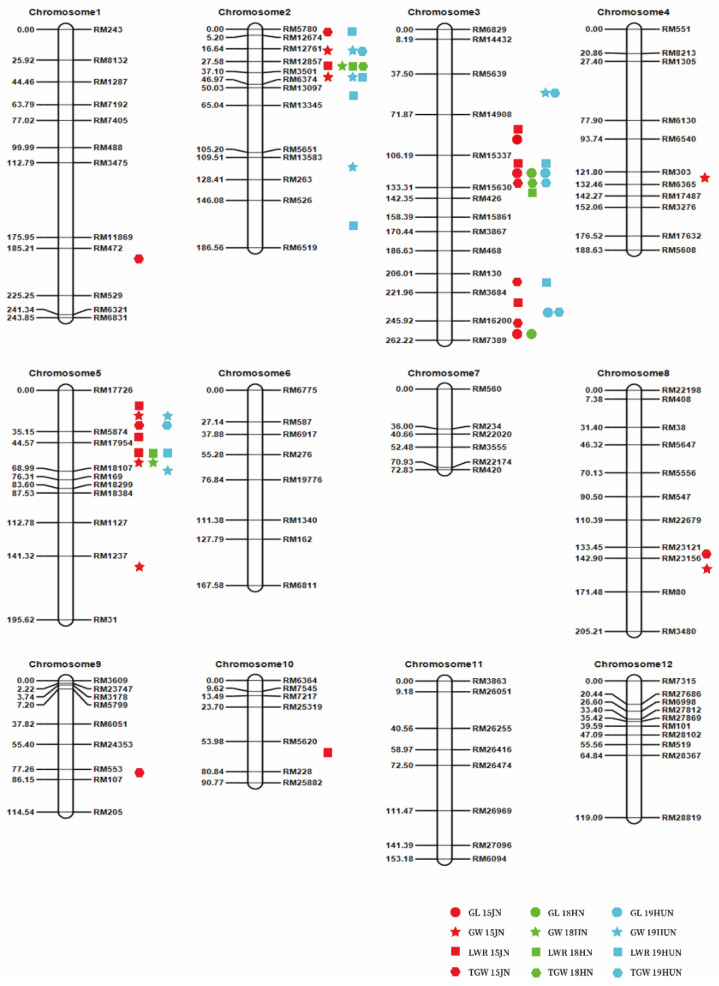
Distribution of identified quantitative trait loci (QTL) for grain size and grain weight on genetic linkage maps in F_2_ populations. The marks denote peak positions of QTL; 15JN, 18HN, and 19HUN represent Jining in 2015, Hainan in 2018 and Hunan in 2019, respectively. Numbers on the left side are the genetic distances between two flanking markers with the unit centiMorgan (cM).

In the F_2:3_ population, up to forty-one QTLs for rice grain size were detected separately in each environment and spread on eight chromosomes (Table S2; Figure 4). Each QTL explained 2.35% to 53.61% of the phenotypic variance.

For GL, four QTLs were discovered on only chromosome 3 across the three tested environments (Table S2; Figure 4). This finding suggested that chromosome 3 is the key chromosome controlling grain length. The major QTL *qGL3.2* overlapped in three environments, accounting for 22.23–53.61% of the phenotypic variation. Another major QTL, *qGL3.4*, explained 12.11% and 11.17% of the phenotypic variation in the two environments, separately.

For GW, 11 QTLs were mapped on chromosomes 2, 3, 4, 5, and 8 (Table S2; Figure 4). Among them, four QTLs were expressed across two environments, and seven QTLs were specific to one location. The proximity of the two QTL (*qGW2.1* and *qGW2.2*) regions indicated that they might represent one QTL detected in different environments. Three major QTLs on chromosome 2 (*qGW2.1*, *qGW2.2* and *qGW2.3*) were identified and explained 9.82–48.61% of the phenotypic variance. Moreover, three major QTLs on chromosome 5 (*qGW5.1*, *qGW5.2* and *qGW5.3*) were detected and revealed high LOD scores ranging from 10.34 to 23.27.

For the LWR, a total of 14 QTLs were found and distributed on four chromosomes, with PVEs of 2.51–33.68% (Table S2; Figure 4). Among them, three QTLs (*qLWR2.2*, *qLWR3.2* and *qLWR5.3*) were significant in multiple environments, and only one QTL, *qLWR5.3*, was identified across all three environments. In a single environment, the largest number of QTLs was identified in Jining in 2015.

For TGW, 12 QTLs were mapped on chromosomes 1, 2, 3, 5, 8, and 9 (Table S2; Figure 4). *qTGW3.2* and *qTGW5.1* were consistently observed in multiple environments, and others were environment-specific QTLs. A major QTL, *qTGW3.2*, was constantly resolved in all three environments, explaining 24.01–33.47% of the phenotypic variance.

It is worth noting that some QTLs were located in nearly the same region (Table S2). The area between RM12674 and RM3501 on chromosome 2 was connected with *qGW2.1*, *qGW2.2*, *qLWR2.2, qTGW2.2* and *qTGW2.3*; and the hereditary effects of these QTLs might all result from the major gene *GW2* and other new minor genes. Combined with the results of whole-genome sequencing, it was found that a 1-bp deletion resulted in a premature stop codon in exon 4 of *GW2*. DNA sequencing also showed this difference in *GW2* between CG and 9311. Based on the sequencing results and EnsemblPlants (http://plants.ensembl.org; accessed on 16 May 2021) data, we predicted the amino acid sequences of GW2 in the coding regions of 9311, CG and Nipponbare (Figure S1). Other major QTLs for GL (*qGL3.3*, *qGL3.4*, *qLWR3.5*, *qTGW3.4* and *qTGW3.5*) were located in the same region as *GL3.3*. We also found base mutation-induced alternative splicing of *OsSK41/OsGSK5* in whole-genome sequencing results. These results are consistent with those previously published [31]. These results also demonstrated the accuracy of the high-throughput sequencing results.

### 2.4. Construct NIL and Analysis of Additive Effects of Some QTLs

**Figure 5:**
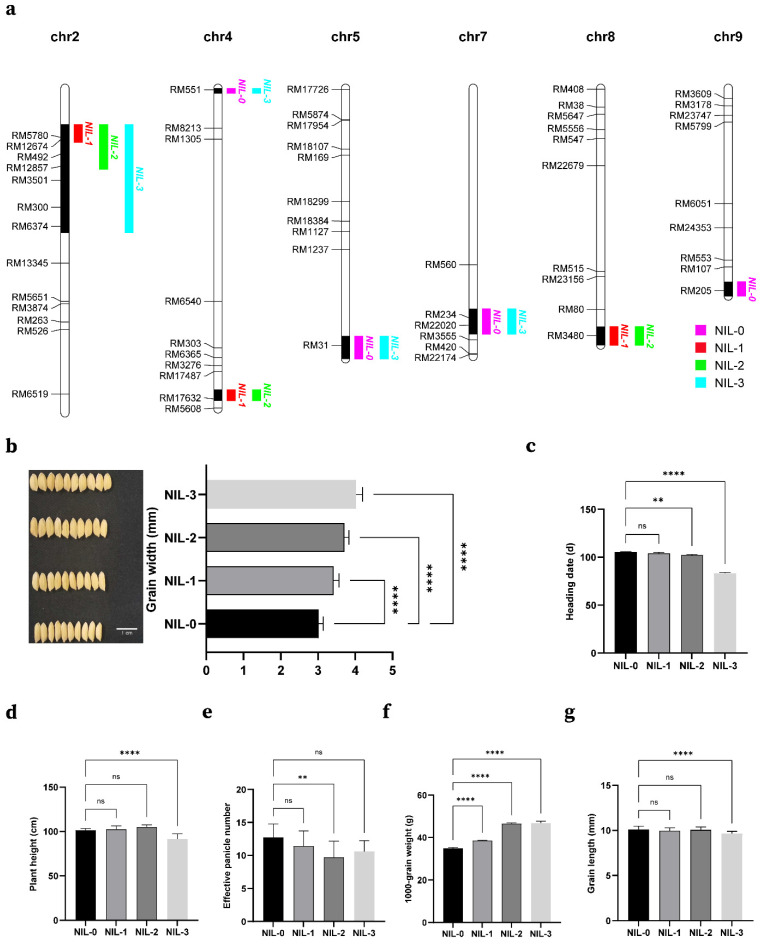
Yield related agronomic traits of the NILs and additive effects of grain width QTLs in NILs. (**a**) The introgression segments of four NILs genotype. (**b**) The additive effect of grain width QTLs, Bar, 1 cm. (**c**–**g**) Yield-related agronomic traits (heading date, plant height, effective panicle number, 1000-grain weight, and grain length) of the NILs. Student’s *t*-test was used to generate *p* values; ns, no significant difference, **** *p* < 0.0001, ** *p* < 0.01.

To assess the adjustment effects of several adjacent QTLs on chromosome 2, we developed four NILs in the BC_3_F_5_ population by marker-assisted selection (Figure 5a). NIL-0 (similar to 9311), NIL-1 (containing *qLWR2.1/qTGW2.1*), NIL-2 (*qGW2.1* and *qGW2.2*), and NIL-3 (*qGW2.1*, *qGW2.2* and *qGW2.3*) had positive additive effects on grain width (Figure 5b,c). Thus, compared to NIL-0, NIL-2 and NIL-3 had significantly increased grain width, by 22.9% and 33.2%, respectively. The 1000-grain weights of the two were almost the same and more than 33% higher than that of NIL-0, which might be due to the slightly longer grain length of NIL-2 compared to NIL-3 (Figure 5f,g). Moreover, we constructed NIL-1 using the tightly-linked molecular markers RM5780 and RM12674. Although the locus in NIL-1 was not detected in grain width alone, it still increased grain width, and we suspected that it might be a minor QTL covered by *GW2*.

### 2.5. Candidate Gene Analysis of the qTGW2.1 Target Region

Since this locus was a minor QTL, we could only perform fine mapping by combining the constructed NIL-1 with high-throughput sequencing. To avoid missing the possible mutation sites, we expanded the screening range and finally selected 5,350,000–5,780,000 bp on chromosome 2 as the candidate interval for *qTGW2.1*. SNPs and InDels existing in the above interval were screened by whole-genome sequencing results, and the genes at the loci causing frameshift, nonsynonymous, or alternative splicing were preferentially selected as candidate genes; 39 genes were finally screened (Table S3).

## 3. Discussion

### 3.1. Strategy: An Efficient and Economical Approach for QTL Mapping

Rice yield is closely related to grain size. A few QTLs of rice grain size have been detected and characterized [35]. Previously, traditional BSA was shown to be an elegant method to detect molecular markers that are closely related to QTLs or target genes for an assigned phenotype [36]. It should be noted that the practicality of DNA markers is the major factor constraining their usefulness. Moreover, genotyping of the bulked DNAs expends considerable energy and money.

In recent years, high-throughput sequencing technologies have been gradually applied to map genes. Combining high-throughput sequencing and BSA has been shown to be an effective and economical method for QTL mapping [37]. Some successful studies have been reported in rice [37,38,39,40].

In this study, we not only used SNPs obtained by high-throughput sequencing technology for primary mapping of grain weight, but also developed the detected InDels into molecular markers, which contributed to marker-assisted selection and fine mapping of the newly discovered QTLs.

### 3.2. Novel QTLs Are Identified for Grain Size

Considering the great difference in grain size between CG and 9311, we used the F_2_ population to map QTLs in different environments. Finally, up to 41 QTLs related to grain size were detected in these populations. We contrasted corresponding and colocalized QTLs with previously reported QTLs for similar or identical traits, according to the physical positions of markers closely associated with the base linkage map. Most of the QTLs detected in our study were found to be located at similar or identical chromosomal regions as previously reported [6,7,10,31,41]. *qGW2.1* and *qGW2.2* affect grain width and are linked to RM12857 (8.6 Mb) and may be identical to *GW2* (8.1 Mb) [6]. *qGL3.3* and *qGL3.4,* which affect grain length, are linked to RM16200 (35.6 Mb) and may be the same as *GL3.3* (35.4 Mb) [31]. We verified these reported major QTLs using whole-genome sequencing results.

Furthermore, comparing the other QTLs in the three regions, we regarded some of them as having the same loci. For instance, *qGL3.2*, *qLWR3.2*, *qLWR3.3* and *qTGW3.2* were mapped to the same location as *OsPPKL1* [10]. *qGW5.1*, *qLWR5.1*, *qLWR5.2* and *qTGW5.1* were mapped to the same location as *GS5* [41]. *qGW5.2*, *qGW5.3* and *qLWR5.3* were mapped to the same location as *GW5* [7].

In addition, we identified two valuable new loci (*qTGW2.1* and *qGW2.3*) and fine mapped one of them. There are many candidate genes related to rice growth and development in *qTGW2.1*. For example, *Os02g0202000* (*OsWR1*) is an ethylene response factor that regulates wax synthesis and drought tolerance [42]. *Os02g0202400* (*OsBT1*) encodes an ADP-glucose transporter protein that controls the synthesis of starch during grain development [43]. *Os02g0202800* encodes the FAR1 domain-containing protein. In Arabidopsis, FAR1 and its homologue FHY3 have crucial functions in plant growth and development [44].

The above QTLs showed stability in different genetic backgrounds and environments and could be used for MAS. The detected alleles are crucial for NILs based on the 9311 genetic background in biodiversity research and molecular breeding. A number of the major QTLs will be further applied for fine mapping, cloning, and analysis of their genetic mechanisms.

### 3.3. Alternative Splicing in the Mutant CG

**Figure 6:**
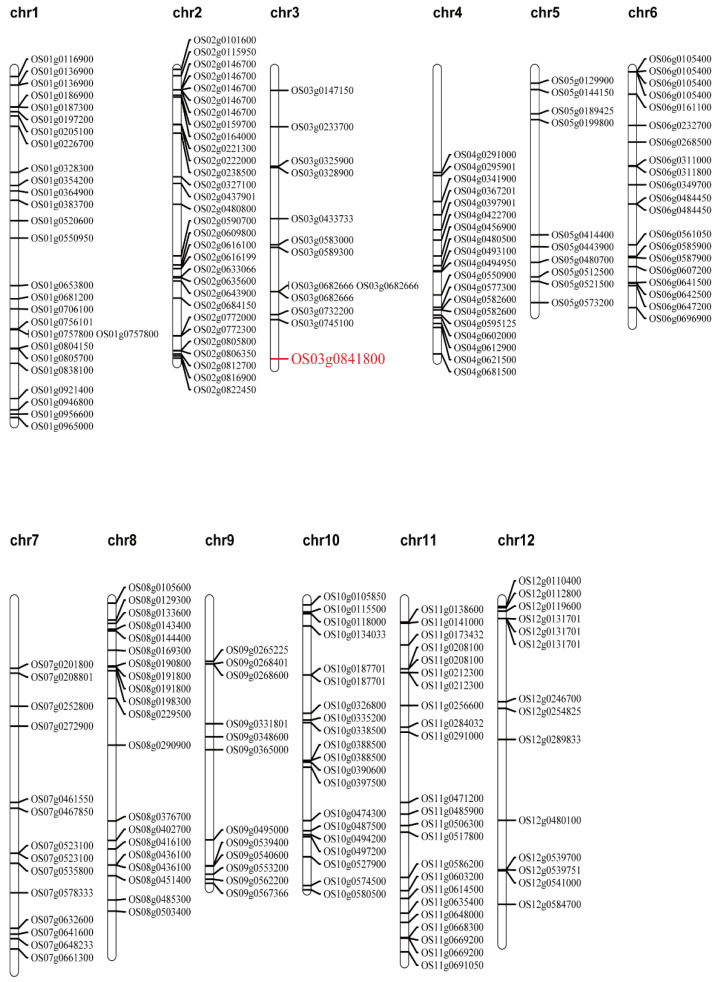
Distribution of identified splicing sites, which compared the whole genome sequencing of CG with the Nipponbare reference genome.

We compared the whole-genome sequencing results with the Nipponbare reference genome and identified 222 splicing sites, including the published gene *OsGSK5* (Figure 6; Table S4). *OsGSK5* can encode a GSK3/SHAGGY-like kinase and negatively affect GL and TGW by regulating cell size and number. Other splicing sites may also have some functions in regulating rice growth and development and responding to stress.

Whole-genome and transcriptome datasets of different rice varieties can serve as a powerful resource for the biological interpretation of trait-related loci, splicing isoform ratios and phenotypic results to help produce high-yielding rice varieties [45]. In future studies, transcriptome datasets of CG can be used to verify these splicing sites. We will use The Rice Annotation Project Database (https://rapdb.dna.affrc.go.jp/; accessed on 22 May 2021) to analyze and predict the function of the genes where these splicing sites reside. Furthermore, the splicing variants between CG, 9311 and Nipponbare were detected by a semiquantitative technique, and their phenotypic effects on different rice varieties were determined.

### 3.4. NIL-3 Is a Possibly Beneficial Resource for Rice Breeding

The results of planting in Hainan showed that the plant height and the heading stage of NIL-3 were significantly different from those of other NILs, which may be due to the presence of a gene near *qGW2.3* that controls plant height and heading stage (Figure 5d,e). In subsequent studies, we will construct a near-isogenic line of this locus, which will be used to locate genes controlling grain width and heading stage.

Yangdao 6 (9311) is a single cropping indica rice variety with disease resistance, high quality, and high yield. The combinations of Yueyou 938, Guangliangyou 6, and Liangyoupeijiu were bred with Yangdao 6 as the male parent, which also had a good quality, high yield, and disease resistance and presented good prospects for popularization and application. These results indicate that a high level of inbred lines might be beneficial for improving the level of hybrid rice breeding. The NIL-3 constructed in this study, not only greatly shortened the growth period of 9311, but also promoted grain shape and yield, which may provide germplasm resources for superior rice breeding and improvement.

## 4. Materials and Methods

### 4.1. Plant Materials

We found a colossal grain rice (CG) of the japonica rice variety Azucena background, which was mutagenized with ethyl methane sulfonate (EMS) to produce a mutant library for screening large grains. After the seed of CG was harvested and planted for multiple generations, its grain phenotype became stable.

For the QTL analysis, an F_2_ population was derived from a cross between the japonica cultivar CG and the indica cultivar 9311 (small, slender grains). The seeds of the F_2_ population were planted in different times and places, while 305 F_2_ plants were harvested in Jining in 2015, 183 F_2_ plants were reaped in Hainan in 2018, and 192 F_2_ plants were successfully self-pollinated in Hunan in 2019, respectively.

From the BC_3_F_5_ generation, four corresponding NILs were screened by molecular marker-assisted selection (MAS).

### 4.2. Phenotypic Measurements and Heritability Estimation

In this study, four grain size traits were measured for all populations: grain length (GL; mm), grain width (GW; mm), length/width ratio (LWR), and 1000-grain weight (TGW; g). Grain length, grain width, length/width ratio, and 1000-grain weight were estimated using 50 grains randomly chosen from grains of individual plants. These traits were calculated by the average of three replicated measurements using Wanshen SC-G automatic seed test analysis and a 1000-grain weight instrument. In addition, descriptive statistics, such as kurtosis and skewness, were procured for F_2_ using the statistical functions of Microsoft Excel 2013, and statistical analysis, such as Student’s t test, was conducted using SPSS statistics 26.0 for Windows (IBM, Armonk, NY, USA).

The broad-sense heritability (*h*^2^*_b_*) for each trait was estimated according to a previously described method [46].

### 4.3. Acquisition and Analysis of BSA Data

We extracted DNA samples from rice leaves and constructed two DNA pools for Pool-seq, according to a previously described method [37,47]. Among them, there was little change in pool size. We constructed two DNA pools (large pool and small pool) using 50 plants from the 2015 Jining experiment. The average sequencing depth of sequencing libraries was over 10× for the parental pools and over 40× for the two descendant pools.

Library construction and analysis of BSA data were performed using Novogene (Novogene, Beijing, China). Detailed sequencing and analysis methods can be found in previous descriptions [37,48,49,50].

### 4.4. DNA Extraction, Molecular Marker Development and PCR Protocol

We extracted total genomic DNA samples from individual plants using the cetyl-trimethyl-ammonium bromide (CTAB) method. Subsequently, the DNA samples were dissolved in double-distilled H_2_O. The quality was determined by 1% agarose gel mixed with ethidium bromide.

The insertion/deletion primers (InDels) were developed using Primer 5.0 based on polymorphisms between the whole-genome sequences of CG and 9311. Primers for amplifying gene fragments were also developed using Primer 5.0. A total of 482 simple sequence repeat (SSR) molecular markers were downloaded from the Gramene website (http://www.gramene.org/archive; accesed on 17 June 2015) and tested with population and parents for polymorphism. However, only 119 of them could be used for QTL mapping and screening target lines for developing near-isogenic lines (NILs).

Total genomic DNA samples were diluted to 100 ng/µL, and PCR amplification was enforced by Vazyme’s protocol with 2× Taq Master Mix (Dye Plus) in a 15-μL reaction volume. Denatured amplified products were then separated on 4% polyacrylamide gels with ethidium bromide.

### 4.5. Construction of Genetic Linkage Maps and QTL Analysis

In light of the genotype of the F_2_ population, QTL IciMapping 4.2 software with the MAP function was used to map QTLs based on 3 years of phenotypic data.

QTL analysis was carried out by the Inclusive Composite Interval Mapping of Additive (ICIM-ADD) module within QTL IciMapping 4.2. The chromosomal location figures of NILs and AS sites were drawn by Mapchart 2.32 [51]. The arguments for detecting putative QTLs and additive QTLs were set according to a previously described method [52].

## 5. Conclusions

Grain size is an important part of quality and harvest traits in rice breeding. Increasing rice yield and ensuring food security are together considered the direction of our future efforts [3]. The QTLs detected in this study will be helpful to elucidate the molecular mechanism of grain size adjustment. In particular, NIL-3 will provide breeding material for superior rice. Furthermore, SNPs identified at splice sites or splicing-related proteins in the present study may play an important role in particularly large phenotypic differences between CG and the other japonica rice.

## Figures and Tables

**Table 1:** Phenotypic values of grain size in F_2:3_ populations.

Trait	Env.	Parents		F_2:3_ Families				*h*^2^*_b_* (%)
		CG	9311	Mean ± SD	Range	Skewness	Kurtosis	
GL (mm)	15JN	13.92 ****	9.66	11.33 ± 0.96	9.18–15.24	0.340	0.431	97.7
	18HN	13.42 ****	9.55	11.35 ± 0.96	9.51–14.75	0.445	0.037	
	19HUN	13.98 ****	9.81	10.59 ± 0.92	8.60–13.91	0.470	0.458	
GW (mm)	15JN	4.24 ****	2.78	3.28 ± 0.38	2.43–4.55	0.214	−0.189	98.5
	18HN	4.22 ****	2.85	3.46 ± 0.33	2.76–4.50	0.542	−0.188	
	19HUN	4.31 ****	2.81	3.05 ± 0.31	2.44–3.95	0.478	0.062	
LWR	15JN	3.29 ***	3.48	3.48 ± 0.39	2.30–4.81	0.182	0.003	93.9
	18HN	3.18 ***	3.36	3.31 ± 0.37	2.42–4.33	−0.007	−0.239	
	19HUN	3.25 ****	3.49	3.50 ± 0.41	2.49–4.67	0.256	0.015	
TGW (g)	15JN	69.25 ****	32.15	42.71 ± 7.57	22.00–64.34	−0.048	−0.303	90.6
	18HN	65.00 ****	32.25	45.23 ± 6.22	31.30–70.10	0.385	0.776	
	19HUN	67.09 ****	31.58	44.83 ± 6.35	28.86–67.50	0.470	0.469	

15JN, Jining 2015; 18HN, Hainan 2018; 19HUN, Hunan 2019. GL, grain length (mm); GW, grain width (mm); LWR, length/width ratio; TGW, thousand-grain weight (g); SD, standard deviation; Env. represents environments; *h*^2^*_b_* represents broad-sense heritability. Student’s *t*-test was used to generate *p* values; *** *p* < 0.001; **** *p* < 0.0001.

**Table 2:** Phenotypic correlation between four grain traits (GL, GW, TGW, and LWR) of the F_2:3_ populations across three environments.

Trait	Env.	GL	GW	TGW	LWR
GL	15JN	1			
	18HN	1			
	19HUN	1			
GW	15JN	0.385 **	1		
	18HN	0.163 *	1		
	19HUN	0.241 **	1		
TGW	15JN	0.740 **	0.631 **	1	
	18HN	0.751 **	0.627 **	1	
	19HUN	0.790 **	0.708 **	1	
LWR	15JN	0.339 **	−0.728 **	−0.114 *	1
	18HN	0.603 **	−0.683 **	0.046	1
	19HUN	0.536 **	−0.682 **	−0.027	1

* *p* < 0.05, ** *p* < 0.01 Env. represents environments; 15JN, 18HN, and 19HUN represent Jining in 2015, Hainan in 2018, and Hunan in 2019, respectively.

## Data Availability

All of the data generated or analyzed during this study are included in this published article.

## Supplementary Materials

The following supporting information can be downloaded at: https://www.mdpi.com/article/10.3390/ijms23073526/s1.
