# Supplementary material for: Identifying QTLs for Grain Size in a Colossal Grain Rice (Oryza sativa L.) Line, and Analysis of Additive Effects of QTLs
Source: Int J Mol Sci. 2022 Mar 24;23(7):3526. doi: 10.3390/ijms23073526 (PMC8998697; doi:10.3390/ijms23073526)
Supplement: Supplementary file 1 [file ijms-23-03526-s001.zip › ijms-1645815-supplementary.pdf]

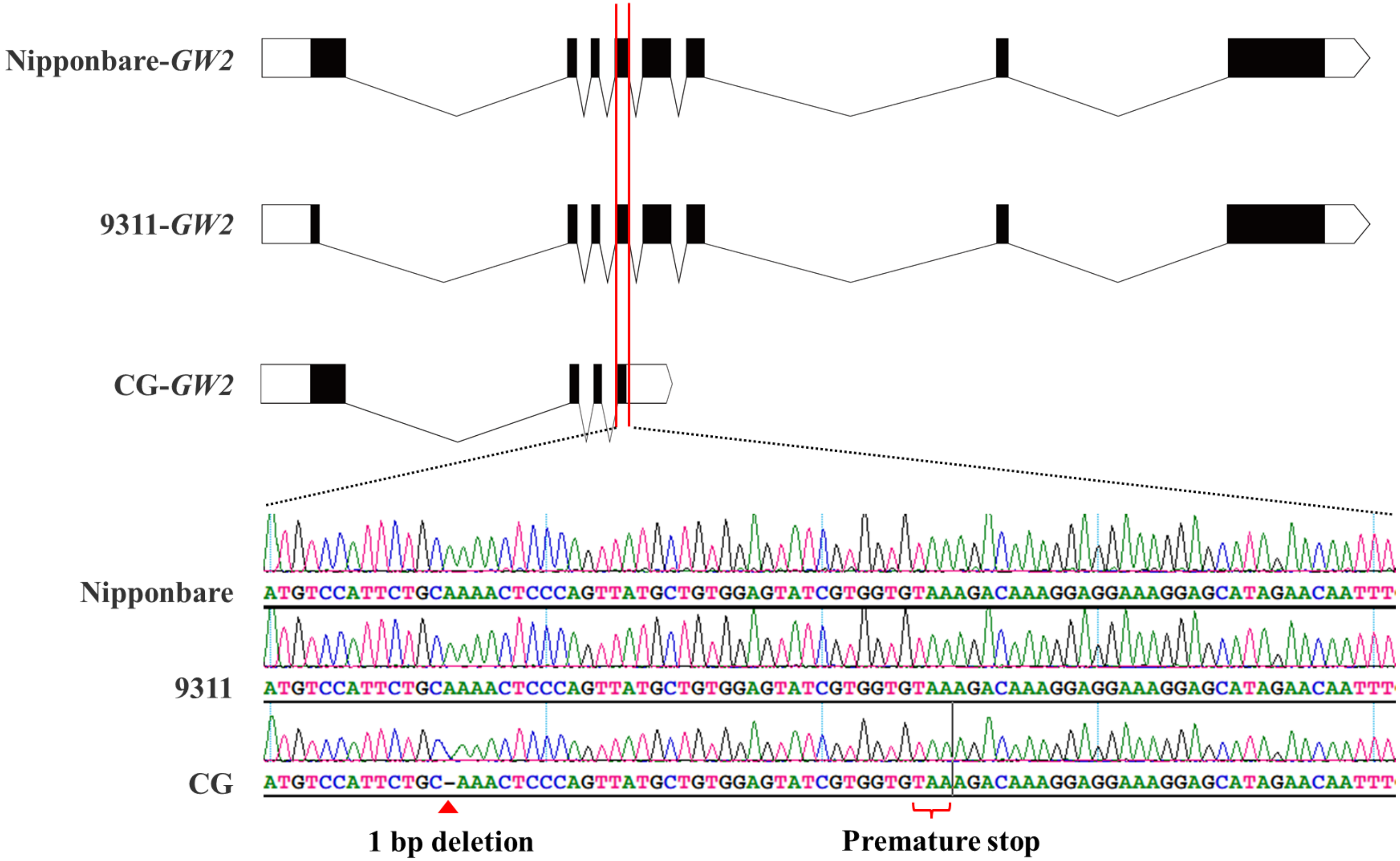

Supplementary Fig. 1 Structure of *GW2* and partial Sequence alignment among Nipponbare, 9311 and CG.
The primers for amplifying the area inside the red line are as follow.
8117284-F: CACACTGCTCAGCCTACACA. 8117284-R: GCATCCTCATTTGTGCTTCT

Supplementary Table 1 Summary of the paired-end sequencing results.

Sample	Clean Reads	Clean Bases (bp)	Clean Q30	GC content (%)	Coverage at least 1X (%)	Average depth
CG	34,835,652	5,225,347,800	90.17	44.36	92.70	13.51×
9311	31,949,878	4,792,481,700	89.85	47.40	89.34	12.47×
B-pool	127,452,818	19,117,922,700	88.01	45.31	96.18	46.01×
S-pool	110,758,536	16,613,780,400	88.91	45.87	96.92	40.39×

B-pool and S-pool are formed by mixing the same amounts of DNA from 50 big grain plants and 50 small grain plants, respectively.

Supplementary Table 2 QTLs detected for grain-related traits in the F_2 population at three environments.

Traits	QTL	Chromosome	Marker interval	15JN				18HN				19HUN				Candidate gene
				LOD	PVE	A	D	LOD	PVE	A	D	LOD	PVE	A	D	
GL	*qGL3.1*	3	RM14908_RM15337	6.16	6.31	-0.44	-0.03									
	*qGL3.2**	3	RM15337_RM15630	24.22	22.23	-0.81	0.13	18.15	53.61	0.97	0.21	33.96	43.56	1.08	−0.02	*OsPPKL1*
	qGL3.3	3	RM3684_RM16200									16.37	15.13	0.54	−0.30	*GL3.3*
	*qGL3.4**	3	RM16200_RM7389	16.37	12.11	−0.56	−0.06	5.87	11.17	0.42	−0.23					*GL3.3*
GW	*qGW2.1**	2	RM12674_RM12857	12.88	9.82	−0.17	−0.06					19.03	14.73	0.18	−0.02	*GW2*
	qGW2.2	2	RM12857_ RM3501					22.46	48.61	0.33	−0.02					*GW2*
	*qGW2.3**	2	RM3501_RM6374	13.30	10.28	−0.17	−0.02					19.40	15.01	0.17	−0.10	
	qGW2.4	2	RM13583-RM526									5.50	3.43	−0.08	−0.01	
	qGW3.1	3	RM5639-RM15337									13.09	18.72	0.20	−0.03	
	qGW4.1	4	RM303_RM6365	3.34	5.51	0.15	0.06									
	*qGW5.1**	5	RM17726_RM5874	11.66	14.44	−0.20	−0.07					23.27	19.35	0.17	−0.03	*GS5*
	*qGW5.2**	5	RM17954_RM18107	10.34	9.82	−0.16	−0.03	13.41	25.28	0.27	0.01					*GW5*

	qGW5.3	5	RM18107_RM169									10.63	7.36	0.09	−0.09	*GW5*
	qGW5.4	5	RM1237_RM31	4.36	3.05	−0.05	−0.11									
	qGW8.1	8	RM23156_RM80	3.11	2.38	−0.06	0.07									
	qLWR2.1	2	RM5780_RM12674									3.96	3.06	−0.12	0.02	
	*qLWR2.2**	2	RM12857_RM3501	21.18	13.86	0.25	−0.02	10.37	27.84	−0.28	0.02					*GW2*
	qLWR2.3	2	RM3501_RM6374									16.20	14.63	−0.26	0.04	
	qLWR2.4	2	RM6374_RM13345									3.00	2.51	−0.03	0.14	
	qLWR2.5	2	RM526_RM6519									3.13	4.63	0.12	0.04	
	qLWR3.1	3	RM14908_RM15337	6.48	5.24	−0.16	−0.04									
LWR	*qLWR3.2**	3	RM15337_RM15630	6.67	6.39	−0.16	0.08					7.95	9.38	0.20	−0.02	*OsPPKL1*
	qLWR3.3	3	RM15630_RM426					4.64	11.37	0.19	0.06					*OsPPKL1*
	qLWR3.4	3	RM130_RM3684									5.62	4.54	0.12	−0.05	
	qLWR3.5	3	RM3684_RM16200	3.88	2.92	−0.12	−0.08									*GL3.3*
	qLWR5.1	5	RM17726_RM5874	7.73	7.48	0.19	0.09									*GS5*
	qLWR5.2	5	RM5874_RM17954	7.84	4.58	0.13	0.08									*GS5*
	*qLWR5.3**	5	RM17954_RM18107	6.50	5.07	0.14	0.11	6.44	19.73	−0.26	−0.01	28.53	33.68	−0.35	0.07	*GW5*
	qLWR10.1	10	RM5620_RM228	4.49	3.55	0.13	−0.04									
	qTGW1.1	1	RM472_RM529	5.38	4.36	−0.69	2.76									
	qTGW2.1	2	RM5780_RM12674	14.31	10.44	−3.18	−0.48									
	qTGW2.2	2	RM12761_RM12857									13.55	10.66	4.02	−0.49	*GW2*
	qTGW2.3	2	RM12857_ RM3501					8.15	16.12	3.68	0.32					*GW2*
TGW	*qTGW3.1*	3	RM5639_RM15337									8.88	14.68	4.49	−0.38	
	*qTGW3.2**	3	RM15337_RM15630	21.71	24.01	−5.03	0.01	11.38	33.47	6.08	−0.08	21.53	24.14	5.49	−0.23	*OsPPKL1*
	qTGW3.3	3	RM130_RM3684	5.91	5.16	−2.47	−0.34									
	qTGW3.4	3	RM3684_RM16200									9.12	6.67	2.42	−1.85	*GL3.3*
	qTGW3.5	3	RM16200_RM7389	9.10	7.16	−2.61	0.10									*GL3.3*

*qTGW5.1**	5	RM17726_RM5874	9.06	6.17	−2.36	0.28					8.20	7.94	2.73	−1.25	*GS5*
qTGW8.1	8	RM23121_RM23156	3.39	2.35	−0.67	−1.97									
qTGW9.1	9	RM553_RM107	5.08	3.55	2.03	0.19									

QTLs identified are named with trait abbreviations followed by the chromosome number;
LOD, $\log_{10}$ of odds ratio;
PVE, percentage of phenotypic variance explained by a single QTL; A, additive effect; D, dominant effect;
* indicates a locus detected in more than one environment.

Supplementary Table 3 Functional annotation of the 39 gene models as harbored in the candidate interval.

	Gene Model ID	Variant	*Position	variation information	Functinal annotation
1	Os02g0203300	frameshift insertion	2:5775769-5775769	exon1:c.2_3insGA:p.M1fs,	Similar to UDP-glycosyltransferase UGT75E3
2	Os02g0195800	nonsynonymous	2:5353652	exon3:c.G277C:p.E93Q,	Similar to Chaperone protein dnaJ 6 (AtJ6) (AtDjC6)
3	Os02g0196600	nonsynonymous	2:5409481	exon7:c.A2112G:p.I704M,	Heavy metal P1B-type ATPase, Cu-transporting ATPase, Control of Cu accumulation in rice grai
4	Os02g0196750	nonsynonymous	2:5413676	exon1:c.G37A:p.D13N,	Hypothetical protein
5	Os02g0196700	nonsynonymous	2:5413676	exon3:c.C1327T:p.R443C,	Similar to Exopolygalacturonase
6		frameshift insertion	2:5413729-5413729	exon3:c.1380_1381insCTAT:p.I460fs,	
7	Os02g0196800	nonsynonymous	2:5417345	exon5:c.G508A:p.V170I,	Similar to Fumarylacetoacetase (Fragment)
8	Os02g0197300	nonsynonymous	2:5451856	exon1:c.C1304T:p.P435L,	Similar to Monoculm1
		nonsynonymous	2:5452717	exon1:c.C443T:p.A148V,	
		nonsynonymous	2:5452733	exon1:c.C427T:p.P143s,	
		nonsynonymous	2:5452766	exon1:c.T394A:p.s132T,	
		frameshift insertion	2:5452776-5452776	exon1:c.383_384insCGGAGAC:p.V128fs,	
		nonsynonymous	2:5452842	exon1:c.C318A:p.F106L,	
		nonsynonymous	2:5452883	exon1:c.G277A:p.D93N,	

		nonsynonymous	2:5452976	exon1:c.G184C:p.A62P,	
		nonsynonymous	2:5452987	exon1:c.G173T:p.G58V,	
9	Os02g0197500	nonsynonymous	2:5466966	exon2:c.A449G:p.K150R,	Regulator of chromosome condensation, RCC1 domain containing protein
10	Os02g0197700	nonsynonymous	2:5472501	exon4:c.C590T:p.A197V,	Di-trans-poly-cis-decaprenylcistransferase-like domain containing protein
		nonsynonymous	2:5472555	exon4:c.T536C:p.V179A,	
11	Os02g0197950	nonsynonymous	2:5482860	exon1:c.C302T:p.P101L,	Hypothetical protein
		frameshift insertion	2:5483025-5483025	exon1:c.136_137insC:p.H46fs,	
		nonsynonymous	2:5483091	exon1:c.A71T:p.D24V,	
		nonsynonymous	2:5483103	exon1:c.A59G:p.H20R,	
12	Os02g0197900	nonsynonymous	2:5488374	exon10:c.T877C:p.s293P,	Conserved hypothetical protein
13	Os02g0198000	frameshift deletion	2:5490692-5490704	exon1:c.1110_1122del:p.370_374del,	Protein of unknown function DUF1677, Oryza sativa family protein
		nonsynonymous	2:5491050	exon1:c.C764T:p.A255V,	
		nonsynonymous	2:5491168	exon1:c.C646A:p.L216I,	
14	Os02g0198200	nonsynonymous	2:5502183	exon1:c.A10G:p.I4V,	EF-Hand type domain containing protein
15	Os02g0198300	nonsynonymous	2:5506787	exon4:c.A280G:p.T94A,	ARF GTPase-activating protein GIT2 (G protein-coupled receptor kinase- interactor 2) (GRK-interacting protein 2) (Cool-interacting tyrosine- phosphorylated protein 2) (CAT2) (CAT-2). Splice isoform 2
16	Os02g0198400	nonsynonymous	2:5517605	exon1:c.T315A:p.D105E,	Similar to predicted protein
17	Os02g0198600	nonsynonymous	2:5528025	exon14:c.C1076A:p.P359H,	Similar to DNA-damage inducible protein DDI1-like
18	Os02g0198700	nonsynonymous	2:5530585	exon7:c.A1999C:p.s667R,	Similar to Subtilisin-like protease
19	Os02g0198800	frameshift insertion	2:5548358-5548358	exon2:c.32_33insGATC:p.L11fs,	Protein of unknown function DUF868, plant domain containing protein
20	Os02g0199200	nonsynonymous	2:5566578	exon1:c.A506G:p.N169s,	Disease resistance protein domain containing protein
		nonsynonymous	2:5567498	exon1:c.T1426C:p.C476R,	
21	Os02g0199300	nonsynonymous	2:5568434	exon7:c.G520A:p.G174s,	Peptidyl-prolyl cis-trans isomerase, FKBP-type domain containing protein

22	Os02g0199500	nonsynonymous	2:5575260	exon1:c.C554T:p.T185M,	Cyclin-like F-box domain containing protein
23	Os02g0199600	nonsynonymous	2:5577009	exon1:c.G113A:p.R38H,	Similar to F-box domain containing protein
		nonsynonymous	2:5577085	exon1:c.T37G:p.C13G,	
24	Os02g0199800	nonsynonymous	2:5591267	exon2:c.C1685T:p.T562I,	Similar to No pollen
		nonsynonymous	2:5593475	exon1:c.A101C:p.Q34P,	
		nonsynonymous	2:5624166	exon1:c.G214A:p.A72T,	
		nonsynonymous	2:5624402	exon2:c.T371C:p.I124T,	
		nonsynonymous	2:5626683	exon7:c.G858T:p.E286D,	
		nonsynonymous	2:5626810	exon7:c.C985T:p.L329F,	
25	Os02g0200900	nonsynonymous	2:5629194	exon3:c.C1255A:p.L419I,	Hypothetical conserved gene
		nonsynonymous	2:5629383	exon3:c.G1066C:p.G356R,	
		nonsynonymous	2:5629502	exon3:c.A947G:p.Q316R,	
		nonsynonymous	2:5629908	exon3:c.T541G:p.L181V,	
		nonsynonymous	2:5630009	exon3:c.G440T:p.R147L,	
26	Os02g0201000	nonsynonymous	2:5641945	exon3:c.G464T:p.R155L,	Conserved hypothetical protein
27	Os02g0201300	nonsynonymous	2:5654836	exon2:c.G410A:p.C137Y,	Conserved hypothetical protein
		nonsynonymous	2:5654846	exon2:c.A400G:p.T134A,	
28	Os02g0201500	nonsynonymous	2:5665924	exon3:c.T995C:p.I332T,	Conserved hypothetical protein
		nonsynonymous	2:5667228	exon2:c.T532G:p.C178G,	
		nonsynonymous	2:5667245	exon2:c.A515G:p.E172G,	
		nonsynonymous	2:5668125	exon1:c.C101T:p.A34V,	
29	Os02g0201800	nonsynonymous	2:5684065	exon1:c.T188C:p.V63A,	Hypothetical gene
		nonsynonymous	2:5684072	exon1:c.G181A:p.E61K,	
		nonsynonymous	2:5684104	exon1:c.T149C:p.I50T,	
30	Os02g0202000	nonsynonymous	2:5688770	exon1:c.C73T:p.R25C,	Ethylene response factor, Transcription factor, Regulation of wax synthesis, Drought toleranc
		nonsynonymous	2:5688849	exon1:c.A152C:p.K51T,	

		nonsynonymous	2:5689207	exon2:c.A389G:p.Q130R,	
		nonsynonymous	2:5710011	exon30:c.G3630A:p.M1210I,	
31	Os02g0202250	nonsynonymous	2:5713568	exon25:c.C3068G:p.A1023G,	Hypothetical conserved gene
		nonsynonymous	2:5716535	exon22:c.G2675A:p.R892Q,	
		nonsynonymous	2:5721529	exon6:c.C818T:p.A273V,	
32	Os02g0202300	nonsynonymous	2:5728655	exon9:c.A1097T:p.Q366L,	Protein of unknown function DUF3414 domain containing protein
33	Os02g0202400	frameshift insertion	2:5737174-5737174	exon1:c.397_398insG:p.S133fs,	ADP-glucose transporter, Plastidic translocator, Starch synthesis during seed developmen
		nonsynonymous	2:5737523	exon2:c.G670A:p.V224I,	
34	Os02g0202600	nonsynonymous	2:5747710	exon2:c.A130C:p.M44L,	Tetratricopeptide-like helical domain containing protein
		nonsynonymous	2:5751103	exon3:c.C1964T:p.A655V,	
		nonsynonymous	2:5752051	exon2:c.T1097C:p.L366s,	
		nonsynonymous	2:5752123	exon2:c.C1025T:p.T342M,	
35	Os02g0202800	nonsynonymous	2:5752391	exon2:c.G757A:p.E253K,	FAR1 domain containing protein
		nonsynonymous	2:5752638	exon2:c.G510C:p.K170N,	
		nonsynonymous	2:5752681	exon2:c.G467A:p.R156Q,	
		nonsynonymous	2:5753501	exon1:c.G29C:p.W10s,	
		nonsynonymous	2:5755777	exon2:c.C905A:p.A302E,	
36	Os02g0202900	nonsynonymous	2:5756230	exon2:c.T452G:p.L151R,	Similar to ring canal kelch
		nonsynonymous	2:5756274	exon2:c.C408A:p.D136E,	
		nonsynonymous	2:5760071	exon1:c.G425A:p.s142N,	
		nonsynonymous	2:5760099	exon1:c.A397G:p.s133G,	
		nonsynonymous	2:5760143	exon1:c.T353C:p.M118T,	
37	Os02g0202950	nonsynonymous	2:5760173	exon1:c.T323C:p.L108P,	Similar to OSIGBa0125M19.6 protein
		nonsynonymous	2:5760413	exon1:c.T83C:p.L28P,	
		nonsynonymous	2:5760432	exon1:c.G64A:p.G22R,	
38	Os02g0203200	nonsynonymous	2:5777250	exon1:c.C55A:p.R19s,	Hypothetical protein

39	Os02g0203300	nonsynonymous<br>frameshift insertion<br>nonsynonymous	2:5777288<br>2:5775769-5775770<br>2:5777288	exon1:c.A17G:p.H6R,<br>exon1:c.2_3insGA:p.M1fs,<br>exon2:c.T1421C:p.V474A,	Similar to UDP-glycosyltransferase UGT75E3

*Position as according to the *Oryza sativa* Japonica reference genome IRGSP-1.0.

Supplementary Table 4 All splicing sites detected from the whole-genome sequencing results.

	Gene name	variation type	chromosome	*position	reference	variation
1	OS01T0116900-00(PAC:OS01T0116900-00:exon2:c.475+2T>C)	splicing	1	939436	T	C
2	OS01T0136900-00(PAC:OS01T0136900-00:exon4:c.1564-2A>C)	splicing	1	1986137	A	C
3	OS01T0136900-00(PAC:OS01T0136900-00:exon4:c.1564-1G>A)	splicing	1	1986138	G	A
4	OS01T0186950-00;OS01T0186900-01(PAC:OS01T0186900-01:exon2:c.774-1T>A)	splicing	1	4647828	T	A
5	OS01T0187300-01(PAC:OS01T0187300-01:exon1:c.56+1G>A)	splicing	1	4668371	G	A
6	OS01T0197200-01(PAC:OS01T0197200-01:exon11:c.1438-1C>T)	splicing	1	5247612	G	A
7	OS01T0205100-01(PAC:OS01T0205100-01:exon9:c.908+2T>C)	splicing	1	5763635	T	C
8	OS01T0226666-00;OS01T0226700-01(PAC:OS01T0226700-01:exon2:c.407-1C>G)	splicing	1	7009914	C	G
9	OS01T0328300-02(PAC:OS01T0328300-02:exon2:c.548+1A>G)	splicing	1	12639698	T	C
10	OS01T0354200-01(PAC:OS01T0354200-01:exon14:c.957-2A>G)	splicing	1	14225180	T	C
11	OS01T0364900-01(PAC:OS01T0364900-01:exon2:c.231-1G>A)	splicing	1	14902433	G	A
12	OS01T0383700-01(PAC:OS01T0383700-01:exon1:c.358+2T>A)	splicing	1	16048474	T	A
13	OS01T0520600-01(PAC:OS01T0520600-01:exon3:c.2545+1G>A)	splicing	1	18537375	C	T
14	OS01T0550900-00;OS01T0550950-00(PAC:OS01T0550950-00:exon3:c.236-1T>C)	splicing	1	20653594	A	G
15	OS01T0653800-02(PAC:OS01T0653800-02:exon18:c.2655-1G>A)	splicing	1	26456531	C	T
16	OS01T0681200-02(PAC:OS01T0681200-02:exon18:c.1842+2T>C)	splicing	1	28062450	A	G
17	OS01T0706100-01(PAC:OS01T0706100-01:exon2:c.1167+1G>T)	splicing	1	29286833	G	T
18	OS01T0756101-00(PAC:OS01T0756101-00:exon3:c.475-1G>A)	splicing	1	31724587	C	T

19	OS01T0757800-01(PAC:OS01T0757800-01:exon5:c.478-2A>T)	splicing	1	31831267	A	T
20	OS01T0757800-01(PAC:OS01T0757800-01:exon5:c.478-1G>T)	splicing	1	31831268	G	T
21	OS01T0804150-00(PAC:OS01T0804150-00:exon1:c.315+1G>C)	splicing	1	34113228	G	C
22	OS01T0805700-01(PAC:OS01T0805700-01:exon11:c.1287+2T>C)	splicing	1	34154723	A	G
23	OS01T0838100-01(PAC:OS01T0838100-01:exon8:c.677-1G>T)	splicing	1	35946060	G	T
24	OS01T0921400-01(PAC:OS01T0921400-01:exon1:c.339+2G>A)	splicing	1	40235589	G	A
25	OS01T0946800-00(PAC:OS01T0946800-00:exon1:c.33+1G>A)	splicing	1	41614193	G	A
26	OS01T0956600-01(PAC:OS01T0956600-01:exon4:c.503-2A>T)	splicing	1	42153407	A	T
27	OS01T0965000-00(PAC:OS01T0965000-00:exon11:c.2807+1G>A)	splicing	1	42552557	C	T
28	OS02T0101600-00(PAC:OS02T0101600-00:exon3:c.303-1G>A)	splicing	2	85264	C	T
29	OS02T0115950-00(PAC:OS02T0115950-00:exon3:c.280+2C>T)	splicing	2	839986	C	T
30	OS02T0146700-02(PAC:OS02T0146700-02:exon3:c.228-2A>T)	splicing	2	2564375	A	T
31	OS02T0146700-02(PAC:OS02T0146700-02:exon7:c.523-1G>C)	splicing	2	2566027	G	C
32	OS02T0146700-02(PAC:OS02T0146700-02:exon8:c.589-2A>C)	splicing	2	2566237	A	C
33	OS02T0146700-02(PAC:OS02T0146700-02:exon9:c.628-2A>T)	splicing	2	2566375	A	T
34	OS02T0146700-02(PAC:OS02T0146700-02:exon22:c.2336+1G>A)	splicing	2	2570135	G	A
35	OS02T0159700-01(PAC:OS02T0159700-01:exon3:c.204+1G>C)	splicing	2	3232811	C	G
36	OS02T0164000-00(PAC:OS02T0164000-00:exon16:c.2202+2T>C)	splicing	2	3442777	A	G
37	OS02T0221300-01(PAC:OS02T0221300-01:exon1:c.1051+2T>C)	splicing	2	6775095	T	C
38	OS02T0222000-00(PAC:OS02T0222000-00:exon3:c.1189-1G>A)	splicing	2	6804733	G	A
39	OS02T0238500-01(PAC:OS02T0238500-01:exon2:c.255+1G>A)	splicing	2	7851921	C	T
40	OS02T0327100-01(PAC:OS02T0327100-01:exon3:c.176+1G>A)	splicing	2	13182999	C	T
41	OS02T0437901-00(PAC:OS02T0437901-00:exon4:c.407-2A>C)	splicing	2	13993220	T	G
42	OS02T0480800-00(PAC:OS02T0480800-00:exon3:c.163-1G>A)	splicing	2	16533720	G	A
43	OS02T0590700-01(PAC:OS02T0590700-01:exon2:c.177+2T>C)	splicing	2	22820485	T	C
44	OS02T0609800-00(PAC:OS02T0609800-00:exon8:c.818+1G>A)	splicing	2	23943842	C	T

45	OS02T0616100-04(PAC:OS02T0616100-04:exon1:c.986+1C>T)	splicing	2	24373831	C	T
46	OS02T0616199-00(PAC:OS02T0616199-00:exon3:c.180-1G>T)	splicing	2	24382366	C	A
47	OS02T0633066-00(PAC:OS02T0633066-00:exon3:c.905-2A>G)	splicing	2	25376801	T	C
48	OS02T0635600-01(PAC:OS02T0635600-01:exon1:c.2542+2T>C)	splicing	2	25490698	T	C
49	OS02T0643900-00(PAC:OS02T0643900-00:exon3:c.786-1G>T)	splicing	2	25889427	G	T
50	OS02T0684150-00(PAC:OS02T0684150-00:exon3:c.711+2T>A)	splicing	2	27978341	A	T
51	OS02T0772000-00(PAC:OS02T0772000-00:exon7:c.1035-2A>G)	splicing	2	32587188	A	G
52	OS02T0772300-01(PAC:OS02T0772300-01:exon6:c.675-2A>G)	splicing	2	32599323	A	G
53	OS02T0805800-01(PAC:OS02T0805800-01:exon7:c.1194+2T>C)	splicing	2	34376561	A	G
54	OS02T0806350-00(PAC:OS02T0806350-00:exon4:c.1157-1G>A)	splicing	2	34408948	C	T
55	OS02T0812700-00(PAC:OS02T0812700-00:exon2:c.267-2A>T)	splicing	2	34802435	A	T
56	OS02T0816900-01(PAC:OS02T0816900-01:exon20:c.2481+1G>T)	splicing	2	35036091	G	T
57	OS02T0822450-00(PAC:OS02T0822450-00:exon2:c.228+1C>T)	splicing	2	35304525	G	A
58	OS03T0147150-00(PAC:OS03T0147150-00:exon2:c.54+2A>G)	splicing	3	2628437	T	C
59	OS03T0233700-00(PAC:OS03T0233700-00:exon3:c.156-2G>A)	splicing	3	7083769	C	T
60	OS03T0325900-00(PAC:OS03T0325900-00:exon2:c.142-1G>T)	splicing	3	11879392	G	T
61	OS03T0328900-02(PAC:OS03T0328900-02:exon3:c.1346+1C>T)	splicing	3	12048716	C	T
62	OS03T0433733-00(PAC:OS03T0433733-00:exon3:c.127-1A>G)	splicing	3	18279701	T	C
63	OS03T0583000-00(PAC:OS03T0583000-00:exon2:c.94+2T>A)	splicing	3	21460946	A	T
64	OS03T0589300-01(PAC:OS03T0589300-01:exon5:c.365-1G>A)	splicing	3	21788354	C	T
65	OS03T0682666-00(PAC:OS03T0682666-00:exon1:c.110+1T>C)	splicing	3	27188179	T	C
66	OS03T0682666-00(PAC:OS03T0682666-00:exon1:c.110+2G>A)	splicing	3	27188180	G	A
67	OS03T0682666-00(PAC:OS03T0682666-00:exon2:c.111-1T>C)	splicing	3	27188196	T	C
68	OS03T0732200-01(PAC:OS03T0732200-01:exon2:c.234+1G>A)	splicing	3	29977789	C	T
69	OS03T0745100-00(PAC:OS03T0745100-00:exon3:c.762-2G>T)	splicing	3	30610028	C	A
70	OS03T0841800-01(PAC:OS03T0841800-01:exon4:c.306+1G>A)	splicing	3	35390422	C	T

71	OS04T0291000-01(PAC:OS04T0291000-01:exon2:c.167-2A>G)	splicing	4	12662563	A	G
72	OS04T0295901-00(PAC:OS04T0295901-00:exon2:c.252-2A>G)	splicing	4	13026794	A	G
73	OS04T0341900-00(PAC:OS04T0341900-00:exon3:c.321+1G>A)	splicing	4	16199684	C	T
74	OS04T0367201-00(PAC:OS04T0367201-00:exon3:c.953-2A>G)	splicing	4	17816991	T	C
75	OS04T0397901-01(PAC:OS04T0397901-01:exon4:c.251-2A>G)	splicing	4	19641680	T	C
76	OS04T0422700-01(PAC:OS04T0422700-01:exon3:c.133-2A>G)	splicing	4	20905052	T	C
77	OS04T0456900-00(PAC:OS04T0456900-00:exon2:c.789-1G>C)	splicing	4	22835680	G	C
78	OS04T0480500-01(PAC:OS04T0480500-01:exon1:c.1309+1G>C)	splicing	4	24033863	G	C
79	OS04T0493100-01(PAC:OS04T0493100-01:exon2:c.159+2T>C)	splicing	4	24656353	A	G
80	OS04T0494950-01(PAC:OS04T0494950-01:exon3:c.584-2A>G)	splicing	4	24747863	A	G
81	OS04T0550900-00(PAC:OS04T0550900-00:exon3:c.128-1G>A)	splicing	4	27595875	C	T
82	OS04T0577300-01(PAC:OS04T0577300-01:exon2:c.281-2A>G)	splicing	4	29111266	A	G
83	OS04T0582600-01(PAC:OS04T0582600-01:exon5:c.1840+2T>C)	splicing	4	29431703	A	G
84	OS04T0582600-01(PAC:OS04T0582600-01:exon5:c.1763-1G>A)	splicing	4	29431783	C	T
85	OS04T0594800-01;OS04T0595125-00(PAC:OS04T0595125-00:exon2:c.41+1G>C)	splicing	4	30064071	C	G
86	OS04T0602000-00(PAC:OS04T0602000-00:exon1:c.1783+1G>A)	splicing	4	30383506	G	A
87	OS04T0612900-01(PAC:OS04T0612900-01:exon1:c.97+2T>G)	splicing	4	31077316	T	G
88	OS04T0621500-00(PAC:OS04T0621500-00:exon1:c.678+2T>C)	splicing	4	31578196	T	C
89	OS04T0681500-01(PAC:OS04T0681500-01:exon5:c.700-2A>G)	splicing	4	34789494	A	G
90	OS05T0129900-02(PAC:OS05T0129900-02:exon4:c.264+1C>T)	splicing	5	1747751	G	A
91	OS05T0144150-00(PAC:OS05T0144150-00:exon3:c.169-2A>T)	splicing	5	2543941	A	T
92	OS05T0189425-00(PAC:OS05T0189425-00:exon3:c.53-2A>T)	splicing	5	5489919	T	A
93	OS05T0199800-01(PAC:OS05T0199800-01:exon3:c.193-1T>G)	splicing	5	6207238	T	G
94	OS05T0414400-02(PAC:OS05T0414400-02:exon5:c.335-1G>A)	splicing	5	20270131	C	T
95	OS05T0443900-01(PAC:OS05T0443900-01:exon1:c.50+2T>C)	splicing	5	21718953	T	C
96	OS05T0480700-01(PAC:OS05T0480700-01:exon1:c.102+2T>A)	splicing	5	23632486	T	A

97	OS05T0512500-01(PAC:OS05T0512500-01:exon3:c.91-1G>T)	splicing	5	25395753	C	A
98	OS05T0521500-01(PAC:OS05T0521500-01:exon2:c.159-2A>G)	splicing	5	25943284	A	G
99	OS05T0573200-00(PAC:OS05T0573200-00:exon1:c.49+1G>A)	splicing	5	28547703	G	A
100	OS06T0105400-02(PAC:OS06T0105400-02:exon20:c.1583-1G>A)	splicing	6	370569	C	T
101	OS06T0105400-02(PAC:OS06T0105400-02:exon19:c.1490-2A>T)	splicing	6	370762	T	A
102	OS06T0105400-02(PAC:OS06T0105400-02:exon18:c.1489+1G>A)	splicing	6	370888	C	T
103	OS06T0105400-02(PAC:OS06T0105400-02:exon16:c.1224-1G>T)	splicing	6	371294	C	A
104	OS06T0161100-00(PAC:OS06T0161100-00:exon2:c.742+2T>C)	splicing	6	3095206	T	C
105	OS06T0232700-01(PAC:OS06T0232700-01:exon2:c.519+2T>C)	splicing	6	6898613	T	C
106	OS06T0268500-00(PAC:OS06T0268500-00:exon2:c.2103+1G>A)	splicing	6	8936915	C	T
107	OS06T0310801-01;OS06T0311000-00(PAC:OS06T0311000-00:exon5:c.263-2A>C)	splicing	6	11841820	T	G
108	OS06T0311800-00(PAC:OS06T0311800-00:exon2:c.408+2T>C)	splicing	6	11889750	A	G
109	OS06T0349750-00;OS06T0349700-00(PAC:OS06T0349700-00:exon5:c.1258-2A>G)	splicing	6	14151281	T	C
110	OS06T0484450-00(PAC:OS06T0484450-00:exon2:c.258+2T>C)	splicing	6	16507688	A	G
111	OS06T0484450-00(PAC:OS06T0484450-00:exon2:c.258+1G>A)	splicing	6	16507689	C	T
112	OS06T0561050-00(PAC:OS06T0561050-00:exon4:c.237+2G>C)	splicing	6	21481237	C	G
113	OS06T0585900-01(PAC:OS06T0585900-01:exon2:c.155+2T>C)	splicing	6	22867298	A	G
114	OS06T0587900-00(PAC:OS06T0587900-00:exon2:c.2074+2T>C)	splicing	6	23050058	A	G
115	OS06T0607200-01(PAC:OS06T0607200-01:exon7:c.304-2A>G)	splicing	6	24144220	T	C
116	OS06T0641500-00(PAC:OS06T0641500-00:exon2:c.744-2A>G)	splicing	6	26093726	A	G
117	OS06T0642500-01(PAC:OS06T0642500-01:exon1:c.93+1G>A)	splicing	6	26155741	G	A
118	OS06T0647200-02(PAC:OS06T0647200-02:exon6:c.2024-1G>A)	splicing	6	26455255	G	A
119	OS06T0696900-00(PAC:OS06T0696900-00:exon3:c.295-2A>G)	splicing	6	29148830	T	C
120	OS07T0201800-00(PAC:OS07T0201800-00:exon1:c.482+2T>A)	splicing	7	5491193	T	A
121	OS07T0208801-00(PAC:OS07T0208801-00:exon2:c.680+1G>A)	splicing	7	5878278	G	A
122	OS07T0252800-01(PAC:OS07T0252800-01:exon2:c.191-1G>T)	splicing	7	8526036	G	T

123	OS07T0273000-01;OS07T0272900-01(PAC:OS07T0272900-01:exon3:c.196-1A>G)	splicing	7	10115984	T	C
124	OS07T0461550-00(PAC:OS07T0461550-00:exon1:c.287+1G>A)	splicing	7	16192679	G	A
125	OS07T0467900-01;OS07T0467850-00(PAC:OS07T0467850-00:exon4:c.290-2A>G)	splicing	7	16660573	T	C
126	OS07T0523100-00(PAC:OS07T0523100-00:exon2:c.4-1G>A)	splicing	7	20257730	G	A
127	OS07T0523100-00(PAC:OS07T0523100-00:exon3:c.258+1G>A)	splicing	7	20258183	G	A
128	OS07T0535800-01(PAC:OS07T0535800-01:exon4:c.1137-2C>A)	splicing	7	21047519	C	A
129	OS07T0578333-01(PAC:OS07T0578333-01:exon3:c.32-1G>A)	splicing	7	23401199	C	T
130	OS07T0632600-01(PAC:OS07T0632600-01:exon19:c.1914+2T>C)	splicing	7	26239786	T	C
131	OS07T0641600-02(PAC:OS07T0641600-02:exon8:c.638+2T>C)	splicing	7	26702805	A	G
132	OS07T0648233-00(PAC:OS07T0648233-00:exon1:c.993+2T>A)	splicing	7	27066557	T	A
133	OS07T0661300-01(PAC:OS07T0661300-01:exon6:c.1273-1G>T)	splicing	7	27887682	G	T
134	OS08T0105600-00(PAC:OS08T0105600-00:exon3:c.322-1G>T)	splicing	8	294544	C	A
135	OS08T0129200-02;OS08T0129300-00(PAC:OS08T0129300-00:exon1:c.419+1G>C)	splicing	8	1645684	G	C
136	OS08T0133600-01(PAC:OS08T0133600-01:exon2:c.242-2A>T)	splicing	8	1914623	A	T
137	OS08T0143400-02(PAC:OS08T0143400-02:exon3:c.304-1C>T)	splicing	8	2388708	G	A
138	OS08T0144400-01(PAC:OS08T0144400-01:exon3:c.592+2T>C)	splicing	8	2513745	A	G
139	OS08T0169300-00(PAC:OS08T0169300-00:exon2:c.120-1G>T)	splicing	8	4053675	G	T
140	OS08T0190800-01(PAC:OS08T0190800-01:exon4:c.226+2T>C)	splicing	8	5319168	A	G
141	OS08T0191800-00(PAC:OS08T0191800-00:exon6:c.714+1G>A)	splicing	8	5381997	C	T
142	OS08T0191800-00(PAC:OS08T0191800-00:exon3:c.456+1G>C)	splicing	8	5383178	C	G
143	OS08T0198300-01(PAC:OS08T0198300-01:exon2:c.14-2A>T)	splicing	8	5681862	A	T
144	OS08T0229601-01;OS08T0229500-01(PAC:OS08T0229500-01:exon2:c.77+2A>G)	splicing	8	7893187	T	C
145	OS08T0290900-01(PAC:OS08T0290900-01:exon3:c.191+1G>A)	splicing	8	11631139	G	A
146	OS08T0376700-01(PAC:OS08T0376700-01:exon4:c.214-1G>T)	splicing	8	17678429	G	T
147	OS08T0402700-01(PAC:OS08T0402700-01:exon1:c.47+2T>C)	splicing	8	19220614	T	C
148	OS08T0416100-01(PAC:OS08T0416100-01:exon4:c.1644+2T>C)	splicing	8	19905228	A	G

149	OS08T0436100-01(PAC:OS08T0436100-01:exon3:c.706-1G>C)	splicing	8	21191398	G	C
150	OS08T0436100-01(PAC:OS08T0436100-01:exon3:c.813+2T>C)	splicing	8	21191508	T	C
151	OS08T0451400-01(PAC:OS08T0451400-01:exon5:c.412-1G>T)	splicing	8	22044546	C	A
152	OS08T0485300-00(PAC:OS08T0485300-00:exon1:c.202+2T>C)	splicing	8	23984129	T	C
153	OS08T0503200-01;OS08T0503400-00(PAC:OS08T0503400-00:exon2:c.21+1G>A)	splicing	8	24868023	C	T
154	OS09T0265225-00(PAC:OS09T0265225-00:exon2:c.53+1G>T)	splicing	9	4935615	C	A
155	OS09T0268401-00(PAC:OS09T0268401-00:exon2:c.426+2T>G)	splicing	9	5140134	A	C
156	OS09T0268600-01(PAC:OS09T0268600-01:exon4:c.981-2G>T)	splicing	9	5150701	C	A
157	OS09T0331801-00(PAC:OS09T0331801-00:exon1:c.124+1G>A)	splicing	9	9918781	G	A
158	OS09T0348600-00(PAC:OS09T0348600-00:exon6:c.1457-1G>A)	splicing	9	10964829	G	A
159	OS09T0365000-00(PAC:OS09T0365000-00:exon2:c.1313+1G>C)	splicing	9	11992115	C	G
160	OS09T0495000-00(PAC:OS09T0495000-00:exon3:c.413-2A>T)	splicing	9	19175978	A	T
161	OS09T0539400-00(PAC:OS09T0539400-00:exon7:c.1027+1G>C)	splicing	9	21241890	G	C
162	OS09T0540600-01(PAC:OS09T0540600-01:exon4:c.894-2A>C)	splicing	9	21279481	A	C
163	OS09T0553200-01(PAC:OS09T0553200-01:exon7:c.489+2T>C)	splicing	9	21919424	T	C
164	OS09T0562200-00(PAC:OS09T0562200-00:exon1:c.424+2A>G)	splicing	9	22333116	A	G
165	OS09T0567366-01(PAC:OS09T0567366-01:exon3:c.393-1G>A)	splicing	9	22650080	G	A
166	OS10T0105850-00(PAC:OS10T0105850-00:exon3:c.445-1G>A)	splicing	10	432936	C	T
167	OS10T0115500-00(PAC:OS10T0115500-00:exon3:c.142+1G>A)	splicing	10	1001940	C	T
168	OS10T0118000-01(PAC:OS10T0118000-01:exon2:c.286+2T>C)	splicing	10	1141117	A	G
169	OS10T0134033-00(PAC:OS10T0134033-00:exon1:c.313+1G>A)	splicing	10	2119222	G	A
170	OS10T0187701-00(PAC:OS10T0187701-00:exon2:c.818+1G>A)	splicing	10	6026716	G	A
171	OS10T0187701-00(PAC:OS10T0187701-00:exon3:c.819-2A>G)	splicing	10	6027164	A	G
172	OS10T0326800-01(PAC:OS10T0326800-01:exon2:c.92-2A>C)	splicing	10	9078897	A	C
173	OS10T0335200-02(PAC:OS10T0335200-02:exon1:c.259+1G>A)	splicing	10	9625071	G	A
174	OS10T0338500-01(PAC:OS10T0338500-01:exon1:c.112+2T>C)	splicing	10	9830963	T	C

175	OS10T0388500-01(PAC:OS10T0388500-01:exon5:c.509-1G>A)	splicing	10	12836427	G	A
176	OS10T0388500-01(PAC:OS10T0388500-01:exon8:c.905-1G>A)	splicing	10	12837091	G	A
177	OS10T0390600-01(PAC:OS10T0390600-01:exon7:c.361-2G>T)	splicing	10	12978910	G	T
178	OS10T0397500-00(PAC:OS10T0397500-00:exon1:c.251+1G>C)	splicing	10	13375630	G	C
179	OS10T0474300-00(PAC:OS10T0474300-00:exon4:c.637+2T>C)	splicing	10	17648679	A	G
180	OS10T0487500-01(PAC:OS10T0487500-01:exon1:c.328+1G>A)	splicing	10	18446479	G	A
181	OS10T0494200-00(PAC:OS10T0494200-00:exon9:c.785+2T>A)	splicing	10	18775927	T	A
182	OS10T0497300-01;OS10T0497200-00(PAC:OS10T0497200-00:exon2:c.580+2T>C)	splicing	10	18935883	A	G
183	OS10T0527900-01(PAC:OS10T0527900-01:exon3:c.1379-2A>T)	splicing	10	20539039	T	A
184	OS10T0574500-00(PAC:OS10T0574500-00:exon2:c.612+1G>A)	splicing	10	22822141	C	T
185	OS10T0580500-00(PAC:OS10T0580500-00:exon5:c.250+1A>G)	splicing	10	23162222	T	C
186	OS11T0138600-00(PAC:OS11T0138600-00:exon3:c.119-1G>T)	splicing	11	1799858	C	A
187	OS11T0141000-01(PAC:OS11T0141000-01:exon3:c.234+2T>G)	splicing	11	1895242	T	G
188	OS11T0173432-00(PAC:OS11T0173432-00:exon3:c.3649+2T>C)	splicing	11	3631498	T	C
189	OS11T0208100-01(PAC:OS11T0208100-01:exon3:c.907-2A>G)	splicing	11	5532850	T	C
190	OS11T0208100-01(PAC:OS11T0208100-01:exon2:c.906+2T>C)	splicing	11	5533095	A	G
191	OS11T0212300-00(PAC:OS11T0212300-00:exon4:c.593+2T>G)	splicing	11	5839795	A	C
192	OS11T0212300-00(PAC:OS11T0212300-00:exon3:c.364+2T>C)	splicing	11	5840138	A	G
193	OS11T0256600-00(PAC:OS11T0256600-00:exon3:c.496-1G>A)	splicing	11	8458415	G	A
194	OS11T0284032-00(PAC:OS11T0284032-00:exon1:c.51+1G>A)	splicing	11	10185437	G	A
195	OS11T0291000-00(PAC:OS11T0291000-00:exon2:c.639+1G>A)	splicing	11	10582260	C	T
196	OS11T0471300-01;OS11T0471200-01(PAC:OS11T0471200-01:exon4:c.408+2T>C)	splicing	11	16207923	A	G
197	OS11T0485900-01(PAC:OS11T0485900-01:exon3:c.83-2A>G)	splicing	11	17131688	T	C
198	OS11T0506300-00(PAC:OS11T0506300-00:exon5:c.577-2A>T)	splicing	11	18044692	T	A
199	OS11T0517800-00(PAC:OS11T0517800-00:exon8:c.841-1G>A)	splicing	11	18567060	C	T
200	OS11T0586200-00(PAC:OS11T0586200-00:exon4:c.326+1G>A)	splicing	11	22173973	C	T

201	OS11T0603200-01(PAC:OS11T0603200-01:exon3:c.1165-1G>T)	splicing	11	23231672	G	T
202	OS11T0614500-02(PAC:OS11T0614500-02:exon3:c.360-2A>T)	splicing	11	23858851	A	T
203	OS11T0635400-00(PAC:OS11T0635400-00:exon2:c.128+1G>A)	splicing	11	25022823	C	T
204	OS11T0648000-01(PAC:OS11T0648000-01:exon9:c.902+2T>C)	splicing	11	25770174	A	G
205	OS11T0668300-00(PAC:OS11T0668300-00:exon10:c.1606+1G>A)	splicing	11	26976705	G	A
206	OS11T0669200-00(PAC:OS11T0669200-00:exon5:c.702+1G>T)	splicing	11	27026110	G	T
207	OS11T0669200-00(PAC:OS11T0669200-00:exon9:c.1321-1G>A)	splicing	11	27027370	G	A
208	OS11T0691100-00;OS11T0691050-00(PAC:OS11T0691050-00:exon3:c.191-1G>A)	splicing	11	28121065	C	T
209	OS12T0110400-01(PAC:OS12T0110400-01:exon3:c.516+2T>C)	splicing	12	563925	T	C
210	OS12T0112800-00(PAC:OS12T0112800-00:exon2:c.270+2T>C)	splicing	12	658148	T	C
211	OS12T0119600-00(PAC:OS12T0119600-00:exon2:c.171-2A>G)	splicing	12	936898	A	G
212	OS12T0131701-00(PAC:OS12T0131701-00:exon2:c.220-2A>C)	splicing	12	1524037	A	C
213	OS12T0131701-00(PAC:OS12T0131701-00:exon3:c.697+1G>A)	splicing	12	1524691	G	A
214	OS12T0131701-00(PAC:OS12T0131701-00:exon4:c.698-2A>C)	splicing	12	1525051	A	C
215	OS12T0246700-01(PAC:OS12T0246700-01:exon2:c.2798-1G>A)	splicing	12	8164981	G	A
216	OS12T0254825-00(PAC:OS12T0254825-00:exon1:c.378+2T>C)	splicing	12	8691212	T	C
217	OS12T0289833-01(PAC:OS12T0289833-01:exon3:c.261-1G>A)	splicing	12	11178108	C	T
218	OS12T0480100-01(PAC:OS12T0480100-01:exon3:c.274-1G>T)	splicing	12	17625030	G	T
219	OS12T0539700-01(PAC:OS12T0539700-01:exon4:c.1487-2A>G)	splicing	12	21564605	A	G
220	OS12T0539751-00(PAC:OS12T0539751-00:exon4:c.413+2T>C)	splicing	12	21569215	T	C
221	OS12T0541000-01(PAC:OS12T0541000-01:exon1:c.894+2T>C)	splicing	12	21638087	T	C
222	OS12T0584700-01(PAC:OS12T0584700-01:exon3:c.127-2A>G)	splicing	12	24329451	T	C

*Position as according to the *Oryza sativa* Japonica reference genome IRGSP-1.0.
